# Effects of Online Video Sport Spectatorship on the Subjective Well-Being of College Students: The Moderating Effect of Sport Involvement

**DOI:** 10.3390/ijerph20054381

**Published:** 2023-03-01

**Authors:** Yi-Hsiu Lin, Chen-Yueh Chen, Yen-Kuang Lin, Chen-Yin Lee, Chia-Yi Cheng

**Affiliations:** 1 Master Program of Sport Facility Management and Health Promotion, National Taiwan University, Taipei 10617, Taiwan; 2 Doctoral Program of Transnational Sport Management and Innovation, National Taiwan Sports University, Taoyuan 33301, Taiwan; 3 Graduate Institute of Athletics and Coaching Science, National Taiwan Sport University, Taoyuan 33301, Taiwan; 4 Graduate Institute of Educational Art and Healing, Mingdao University, Changhua 52345, Taiwan; 5 Department of Recreation and Leisure Industry Managemnt, National Taiwan Sport University, Taoyuan 33301, Taiwan

**Keywords:** online video sport spectatorship, subjective well-being, sport involvement, mental health, college student

## Abstract

Individuals’ engagement in sports and sport-related entertainment is reported to improve their subjective well-being (SWB). We investigated whether online video sport spectatorship (OVSS) enhances the SWB of college students and whether sport involvement moderates the correlation between OVSS and SWB. For this, a pretest–posttest experimental design with a 3-week-long intervention (OVSS) was adopted. Two groups, intervention and control, were formed. The findings revealed that OVSS improved SWB (*p* = 0.017). Furthermore, sport involvement moderated the correlation between OVSS and SWB (*p* = 0.024). Specifically, participants with high levels of sport involvement in the intervention group exhibited better SWB (M = 5.51) than did the corresponding controls (M = 4.69). By contrast, among participants with low levels of sport involvement, only the intervention group showed improved SWB; however, it remained unchanged in the control group. The present study enriches the relevant literature and provides empirical evidence for the psychological benefits conferred by OVSS. Our findings may serve as a reference for designing interventions aimed at improving individuals’ overall quality of life.

## 1. Introduction

Subjective well-being (SWB), a common goal pursued by many nations, is crucial for young adults [1]. Mental health problems have become a growing concern among college students [2]. Empirical evidence has indicated that the effects of positive mental states outweigh those of the absence of negative mental states in the prediction of morbidity and mortality [3]. Leisure, recreation, sports, and physical exercise may improve the SWB of college students and resolve their mental health problems. Thus, the effects of leisure and sport activities on the SWB and mental health problems of college students warrant further investigation.

Empirical evidence has identified sport participation as a predictor of SWB [4]. Sport participation promotes SWB in youth [5]. Regarding the attitudes of individuals engaged in various sports, playful attitudes exert stronger positive effects on SWB than do competitive attitudes [6]. Physical exercise is positively correlated with SWB in college students [7]. Engagement in sport activities also has robust associations with SWB [8].

A stream of research has focused on the correlation between sport game consumption and SWB. Older adults attending sport games exhibit improved SWB [9]. Furthermore, sport media viewing (SMV) is positively correlated with the viewers’ SWB [10]. Passive sport participation, such as watching sports on television, is positively correlated with happiness [11]. A study conducted in college students revealed that the use of social media is positively correlated with the students’ SWB [12]. Despite empirical evidence indicating that sport participation (both active and passive) improves the SWB of college students, the effects of watching sport events online on the SWB of these students remain to be investigated. Studies must investigate not only the causal relationship between SMV and SWB but also the changes in the viewers’ baseline SWB after a relevant intervention [13]. Earlier empirical studies on the relationship between SMV and SWB adopted a nonexperimental design [4,13,14], which may be unsuitable for determining causal relationships. Furthermore, very few studies have been conducted to explore the correlation of SWB or global well-being with SMV [13,14,15], which warrants further research. Longitudinal and experimental designs are suitable for identifying the predictors and outcomes associated with SWB [16]. Given the aforementioned background, in the present study, we investigated the effects of online video sport spectatorship (OVSS) on the SWB of college students. OVSS in the current study describes individuals’ sport viewing behavior through online videos in a YouTube channel. An experimental design including an intervention was adopted in our study.

Sport involvement is reported to influence individuals’ SMV behaviors [17]. For instance, spectatorship enhances individuals’ intention to follow social media channels if they highly appreciate such a sport [18]. A sport itself and the relevant emotionalized media may evoke individuals’ emotions when they watch sports [19]. A study indicated inconsistency in the correlation between SMV and SWB, suggesting that there are moderating effects for relevant variables [13]. The level of sport involvement not only influences behavioral decisions but also moderates the correlations between the variables associated with behavioral decisions [20]. Thus, to better understand the correlation between OVSS and SWB in the present study, we investigated the moderating effect of sport involvement on this correlation.

The present study has three primary contributions. First, the adoption of an experimental design with a longitudinal intervention bridged the relevant research gap; as mentioned, earlier studies have either focused on comparing baseline and post intervention SWB variables, or they have lacked an intervention. Second, our findings provide empirical evidence for the moderating effect of sport involvement on the correlation between OVSS and SWB. Third, this study enriches the relevant literature and provides insights for college students aiming to improve their SWB.

## 2. Hypothesis Development

### 2.1. SWB

Diener defined SWB as individuals’ assessment of their overall quality of life on the basis of self-defined criteria [21]. SWB further refers to individuals’ affective and cognitive assessments of their lives on the basis of their hedonic experiences [22]. According to hedonic treadmill theory [23], an individual’s psychological state comprises the baseline and temporal fluctuations of SWB. The baseline component of SWB is highly correlated with individuals’ disposition, whereas the temporal component is dependent on their experiences [13]. Furthermore, SWB may be managed and malleableized by subjecting individuals to purposeful interventions or ensuring their frequent engagement in positive activities [24,25,26]. Sport participation, sport event attendance, and SMV may confer utilitarian and hedonic benefits. Thus, individuals’ affective and cognitive assessments of their lives on the basis of their hedonic sport experiences must be explored.

### 2.2. Sports and SWB

The correlation between sports and SWB has attracted considerable academic attention [27]. Specifically, SMV behavior is associated with SWB. Sport participation and consumption are positively correlated with short- and long-term SWB [4,13]. Furthermore, leisure time physical activities are positively correlated with life satisfaction [28]. Compared with studies on sport participation and attendance, those on online sport spectatorship are limited. SMV-related hedonic experiences are positively correlated with SWB [14]; the positive affect due to SMV leads to perceived enjoyment [15]. Despite the passive nature of media consumption, as described in leisure studies [29], SMV was reported to generate happiness and excitement [13,30]; this observation indicates that SMV is a positive predictor of SWB. To overcome the limitation related to the lack of studies focusing specifically on SWB and SMV, Kim and James [13] investigated the correlation between SMV and short- and long-term SWB. Despite empirical evidence indicating a correlation between SMV and SWB, the rationale for a possible causal relationship is insufficient. Furthermore, watching sports (one-time engagement) was identified as a positive predictor of short-term SWB [31]. Considering the aforementioned findings, we hypothesized that OVSS improves the SWB of college students (Hypothesis 1 [H1]).

**H1.**  *OVSS improves the SWB of college students*.

### 2.3. Sport Involvement as a Moderator

Involvement refers to individuals’ perceived relevance of an object based on their inherent needs, values, and interest [32]. It is manifested by the orientation of individuals toward the objects of their interest. In the context of sports, sport involvement refers to the level of importance individuals attach to sport participation and consumption [17]. Sport involvement influences hedonic SMV [33]. Leisure engagement is reported to moderate the perceived negative effect of sport events on individuals’ SWB; the perceived negative effect is buffered when the level of leisure engagement is low, but this effect is nonexistent when the level of leisure engagement is high [27]. In leisure studies, planned leisure activities were demonstrated to enhance SWB and facilitate the development of a positive attitude toward daily life [34]. SMV is a planned leisure activity; thus, it can enhance SWB. However, a study conducted among college students claimed that leisure engagement primarily reduces depression rather than increasing happiness [35]. The inconsistency in the findings of earlier studies indicate the influence of potential moderators on the correlation between SMV and SWB. An empirical study revealed that sport involvement moderates the correlation between persuasion strategies and attitudes toward the corporate social responsibility initiatives adopted by sport organizations; persuasion strategies are effective only when sport involvement is low [36]. Although this finding is not directly related to SWB, it suggests that individuals are sensitive to interventions when their sport involvement is relatively low. SMV is both positively and negatively associated with SWB [13]; a moderator may explain the inconsistent findings. Poiesz and Bont [20] reported that involvement may influence behavioral decisions and even moderate the correlations between the variables associated with such decisions. Premising on their study, we hypothesize that sport involvement would moderate the correlation between OVSS and SWB (Hypothesis 2 [H2]).

**H2.**  *Sport involvement would moderate the correlation between OVSS and SWB*.

## 3. Participants and Methods

### 3.1. Study Design

This experimental study with a pretest–posttest design included college students aged >20 years. A pilot study comprising a total of 50 college students was conducted to identify the favorite sports of the target population. The top three favorite sports identified were basketball (n = 40; 80%), baseball (n = 28; 56%), and volleyball (n = 23; 46%).

### 3.2. Sample Size Calculation

The minimum required sample size, which was based on the interdependence of statistical power (alpha), was calculated using PROC POWER in SAS (version 9.4). The effect size was determined according to the method of Stieger et al. [37], who investigated the effects of watching the game during the 2014 World Cup through a smartphone-based app on SWB. The spectators show greater degree of SWB (M = 81.2) compared with those who did not watch a game (M = 68.3) for the game of Germany versus Portugal. Thus, a sample size of 30 per group was needed to ensure adequate statistical power (0.80) with a type I error of 5%.

Post-hoc power analysis was performed to estimate the obtained power. In this study, a linear mixed model was conducted as the primarily statistical model analysis. Thus, we used the analysis results obtained from the linear mixed model analysis for SWB to estimate the effect size and the obtained power. This study has an adequate power of greater than 0.80 under these assumptions: the regression coefficient for the linear mixed model of 0.987, number of predictors in the 7-predictor total effect model (Time, OVSS, involvement, Time x OVSS, OVSS × involvement, Time x involvement, Time × OVSS × involvement), a probability level of 0.05, and a sample size of 54.

### 3.3. Experimental Procedure and Implementation

This study included a total of 60 college students. A total of 30 participants were each randomly assigned to an intervention group and a control group. Participants were instructed to watch online videos for 20 min per week for 3 weeks. For the intervention group, the content of the aforementioned online videos was sport games excerpted from YouTube. This group watched online videos related to basketball, baseball, and volleyball in weeks 1, 2, and 3, respectively [38]. The adoption of online videos of animals (completely unrelated to sport games) for the control group was derived from the work of Folkvord et al. [39].

Before the intervention, all participants were required to complete an SWB measurement questionnaire; their pretest scores were recoded. After 3 weeks of intervention, participants were requested to complete the same questionnaire to record their posttest scores. Although this study initially included a total of 60 participants, 6 dropped out because of their inability to watch videos as instructed. Thus, the final sample comprised a total of 54 participants (men, 57.4%). Consequently, the intervention and control groups comprised 30 and 24 participants, respectively. The overall level of sport involvement, SWB evaluated at baseline, and SWB evaluated at posttest were 4.5 (standard deviation (SD) = 1.52), 4.9 (SD = 1.22), and 5.3 (SD = 1.18), respectively (Table 1).

### 3.4. Study Protocol

Participants in the intervention and control groups were invited into two separate rooms to watch the corresponding online videos. Subsequently, they completed the posttest questionnaire. This study was approved by the Research Ethics Committee of National Taiwan University. Participants were well informed about the study objectives, procedures, and potential risks and benefits; they could drop out of this study at any time. At the end of week 3, participants completed an online survey.

### 3.5. Measurements

The study questionnaire comprised items that could be categorized into three broad categories: demographics, SWB, and sport involvement. The items on demographics were related to participant sex and prespecified identification number. The SWB category comprised the following two items: *I am happy with my life*, and *In most ways, my life is close to perfect* [40]. The level of sport involvement was measured using the method of Kyle et al. [41]. This category comprised a total of 15 items, which could be subcategorized into the following 5 subdimensions: attraction, centrality, social bonding, identity affirmation, and identity expression. Table 2 summarizes items of this questionnaire. The responses were scored on a 7-item Likert scale with the endpoints ranging from 1 (*strongly disagree*) to 7 (*strongly agree*).

### 3.6. Data Analysis

Means and SD values were calculated to evaluate the central tendency and variation in continuous variables. Cronbach’s alpha and factor analysis were used to evaluate the reliability and validity of the questionnaire responses. The criterion for determining the validity of questionnaire items was the evaluation of dimensionality in terms of factor loadings, eigenvalues, and scree plots.

To investigate whether the pre–post changes in participants’ SWB was correlated with OVSS (H1), a linear mixed model was adopted. This approach is more flexible than the traditional repeated measures analysis of variance because it does not require the sphericity of covariance structure. In addition, this model enables the construction of various covariance matrices, including unstructured, first-order autoregressive, and compound symmetry matrices. We further investigated the moderating effect of sport involvement on the aforementioned correlation. Depending on the varying levels of sports involvement, the intervention was expected to differentially influence SWB. To longitudinally evaluate SWB, the linear mixed model was used to investigate the three-way interaction among time, OVSS, and sport involvement. Participants were regarded as a random effect in this model. This model was used previously for identifying the moderators of treatment outcomes in repeated measures analyses [42,43,44].

In the present study, sport involvement represented a categorical variable. Participants were classified into groups with high and low levels of sport involvement on the basis of their mean scores on the study questionnaire. An independent *t* test was performed to confirm effective grouping before regarding sport involvement as a categorical variable in the linear mixed model.

## 4. Results

### 4.1. Results of H1 Testing

**Figure 1:**
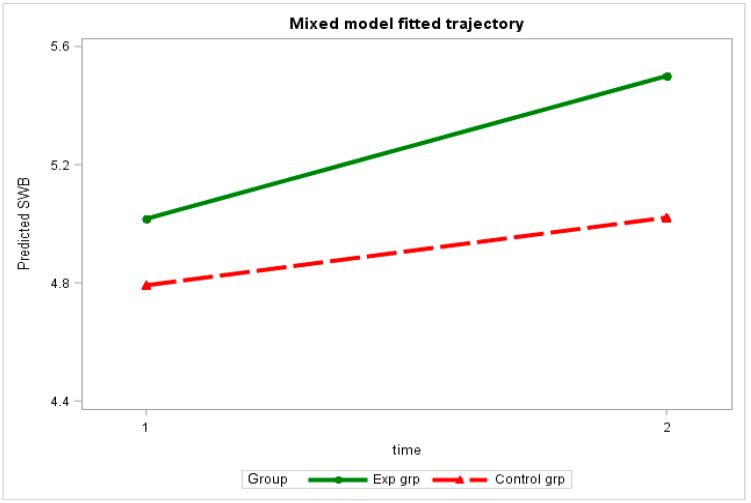
Mixed model fitted trajectory by group.

A mixed 2 × 2 × 2 design was used to test H1. Specifically, we investigated whether OVSS could be regarded as a between-subject variable. Sport involvement was regarded as a between-subject variable, whereas time was considered to be a within-subject variable (before and after the 3-week-long intervention). The results of the linear mixed model revealed a significant correlation between time and OVSS (*p* = 0.017). Participants’ posttest SWB scores were significantly higher than their pretest scores (*p* = 0.001; Table 3). The effect of OVSS on SWB increased gradually (Figure 1), suggesting that OVSS enhances SWB, which supported H1.

### 4.2. Results of H2 Testing

**Figure 2:**
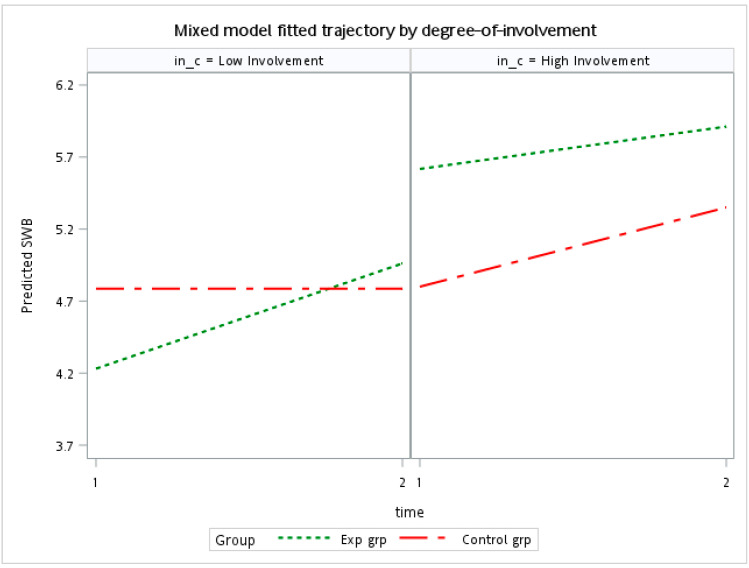
Mean subjective well-being scores versus the levels of sport involvement.

To test H2, a linear mixed model was adopted with time (pre- and posttest) as a within-subject variable. In this analysis, OVSS and sport involvement were regarded as between-subject variables. High levels of sport involvement exerted significant main effects on SWB (*p* = 0.002). The mean SWB scores of participants with high levels of sports involvement was 5.51, which was higher than that of participants with low levels of sport involvement (4.69). A nonsignificant two-way correlation was identified between time and sport involvement, which indicated that sport involvement was not correlated with the changes in SWB after the intervention. As expected, a significant three-way interaction was noted among time, OVSS, and sport involvement (*p* = 0.024; Table 2). Similarly, sport involvement was significantly correlated with OVSS (*p* = 0.033). The direction of the correlation supported H2. Participants with high levels of sport involvement in the intervention group consistently exhibited higher SWB scores than did the corresponding controls. However, among participants with low levels of sport involvement, the mean SWB scores of the intervention group increased after the intervention, but those of the control group remained unchanged (Figure 2). Together, the results indicate that sport involvement moderates the correlation between OVSS and SWB, supporting H2.

## 5. Discussion

### 5.1. Theoretical Implications

In the present study, we investigated the correlation of OVSS with the SWB of college students and the moderating effect of sport involvement on this correlation. The experimental design adopted in our study facilitated causal inferences, thus, helping us overcome the previous limitation that only nonexperimental studies reported a causal relationship between SMV and SWB. In addition, the longitudinal 3-week-long intervention enabled us to evaluate the changes in participants’ SWB due to the intervention. Thus, our findings provide comprehensive information on SWB. Furthermore, the moderating effect of sport involvement on the correlation between OVSS and SWB may explain the previous inconsistent findings. The present study adds to the empirical evidence for the effects of SMV (specifically, online sport spectatorship) on the mental health of college students. We further clarified the moderation effect of sport involvement on the variables of interest.

### 5.2. OVSS and SWB

The SWB of our participants improved after the intervention. This finding is consistent with those of relevant studies on the association of SWB with sports. For instance, SMV is positively correlated with individuals’ SWB due to hedonic experiences from sports activities [12]. SMV may induce a positive effect, such as happiness and excitement [30]. This finding supports that SMV is positively correlated with short- and long-term SWB [13]. However, Kim and James [13] averred that whether SMV is beneficial for SWB remains equivocal. Our findings corroborate the positive effect of SMV (OVSS) on SWB. The findings of this study expand our knowledge regarding the effect of SMV beyond a so-called *one-engagement* sport-watching experience. The findings of this study are consistent with empirical evidence that sport spectating positively predicts SWB effect [31].

### 5.3. Moderating Effect of Sport Involvement

We identified a moderating effect of sport involvement on the correlation between OVSS and SWB. Participants with high levels of sport involvement in the intervention group exhibited better SWB than did the corresponding controls. By contrast, only participants with low levels of sport involvement in the intervention group exhibited improved SWB; however, the SWB of the control group remained unchanged. The moderating effect of sport involvement indicates that OVSS is more beneficial for individuals with lower levels of sport involvement than for those with higher levels of sport involvement. This finding is similar to that of Wu et al. [27], in which leisure engagement was found to moderate the indirect effects of the perceived negative effect of sport events on individuals’ support for Macau Grand Prix via SWB. The indirect effects were prominent only for lower levels of leisure engagement. This finding suggests that individuals with high levels of sport engagement tend to attach high levels of relevance, interest, and importance to sports, such that they are likely to support sport events; this, in turn, may buffer the effects of the perceived negative effect of sport events on individuals’ support for sport events through SWB. By contrast, participants with low levels of leisure engagement showed a tendency to attach lower levels of relevance, interest, and importance to sports, which failed to buffer the effects of the negative perceived effect of sport events on individuals’ support for sport events via SWB.

The rationale of Wu et al. [27] for the moderating effect of leisure engagement may explain the moderating effect of sport involvement on the correlation between OVSS and SWB. In the present study, college students with higher levels of sport involvement in the intervention group exhibited improved SWB compared to the corresponding controls. The higher levels of sport involvement of students might have improved their SWB and positive attitudes toward daily life [34]. By contrast, college students with low levels of sport involvement who attached low levels of relevance, centrality, and importance to sports exhibited improved SWB after the intervention. This might be because these students gained hedonic experiences from the intervention, which, in turn, enhanced SWB. Our finding regarding the moderating effect of sport involvement is consistent with the that of Chen and Lin [36], in which persuasion strategies were found to be effective only in individuals with low levels of sport involvement. Although Chen and Lin [36] used individuals’ attitude toward the corporate social responsibility activities adopted by sport organizations as a dependent variable instead of SWB, the implications of their study are conceptually and logically applicable to the present study. Individuals with high levels of sport involvement often exhibit stronger attitudes toward their favorite sports teams than do those with low levels of sport involvement. Unsurprisingly, persuasion information or messages may be more effective for individuals with low levels of sport involvement than for those with high levels of sport involvement. Moreover, the moderating effect of sport involvement provides scientific evidence for the previous inconsistent correlations between SMV and SWB [13]. Earlier studies have indicated the need for studies focusing on assessing not only the long-term SWB but also the changes in SWB after a relevant intervention [13]. Together, the results indicate that sport involvement moderates the correlation between OVSS and SWB, which is in line with the notion that involvement moderates the correlations between the variables associated with behavioral decisions [20].

### 5.4. Practical Implications

The findings of the present study indicate that OVSS is effective in improving the SWB of college students. Leisure constraints, including interconstraints, intraconstraints, and structural constraints, may deter college students from attending sport events or participating in sport activities, which may prevent them from experiencing improved SWB. In this context, OVSS may improve SWB. College students have easy access to the Internet and smartphones; thus, interventions based on OVSS are feasible. College students with low levels of sport involvement who received the intervention exhibited improved SWB. Students with high levels of sport involvement who received the intervention showed better SWB than did those who did not receive the intervention. Thus, the development of sport involvement through continual sport-related stimuli may represent an effective strategy for improving SWB. The escalator concept in sport marketing suggests that the nonconsumers of sport media can be converted into consumers (light, medium, and heavy consumers) on the basis of their levels of sport involvement and consumption. Thus, individuals’ use of social media may increase their levels of sport involvement.

### 5.5. Limitations and Future Directions

The present study has some limitations. The dropout of six participants created an inconsistency between the intervention and control groups. Despite the provision of incentives, this dropout situation could not be avoided. Future studies must consider the possibility of sample churn when calculating the sample size. Furthermore, the requirement of repeated measurements made it challenging for us to recruit a large number of participants. The limited sample size might not have been sufficient for evaluating questionnaire validity through factor analysis. Future studies are warranted to recruit a sufficient number of participants. In addition, the effects of moderators other than sport involvement, such as sport team identification, may be investigated in future studies. The games were not live, so we cannot exclude the possibility of the effect of games that were not live on participants’ subjective well-being. Therefore, future studies may consider comparing students who watched sports games (live) with those who did not and assess the effect on subjective well-being. Finally, the constraints of this study hindered the adoption of counterbalance measure design. Researchers may consider adopting counterbalance measure design in future studies.

## 6. Conclusions

Mental health is a crucial matter, particularly among college students. Our findings suggest that OVSS positively influences SWB in these students. SWB may be enhanced by increasing the levels of sport participation, sport consumption, and OVSS. Owing to the advancement and accessibility of information technology, OVSS has become a key avenue for receiving sport-related news and watching sports. We identified a causal relationship between OVSS and SWB and clarified the moderating effect of sport involvement on this relationship. Our study enriches the literature on sport and mental health. OVSS may be adopted as a strategy to continually improve SWB and increase sport involvement levels in college students. Our findings may help design interventions aiming at improving individuals’ overall quality of life.

## Figures and Tables

**Table 1:** Descriptive Statistics of the Study Variables.

	Experiment Group	Control Group	Total	*p*-Value
	(n = 30)	(n = 24)	(n = 54)	
Gender				
Male	17 (56.7%)	14 (58.3%)	31 (57.4%)	0.9020
Female	13 (43.3%)	10 (41.7%)	23 (42.6%)	
IN, Mean (SD)	4.8 (1.37)	4.1 (1.64)	4.5 (1.52)	0.11121
SWB at T1, Mean (SD)	5.0 (1.13)	4.8 (1.34)	4.9 (1.22)	0.50581
SWB at T2, Mean (SD)	5.5 (0.97)	5.0 (1.38)	5.3 (1.18)	0.14101

IN, involvement; SD, standard deviation; SWB, subjective well-being.

**Table 2:** Items of the Questionnaire Used in the Present Study.

Items	M	SD
Subjective well-being (pretest)		
1. I am happy with my life.	4.96	1.23
2. In most ways, my life is close to perfect.	4.87	1.30
Satisfaction well-being (posttest)		
1. I am happy with my life.	5.24	1.21
2. In most ways, my life is close to perfect.	5.33	1.23
Sport involvement		
*Attraction*		
1. Sport is one of the most enjoyable things I do.	4.69	1.74
2. Sport is very important to me.	4.31	1.83
3. Sport is one of the most satisfying things I do.	4.39	1.71
*Centrality*		
4. A considerable portion of my life revolves around sports.	4.48	1.82
5. Sports play a central role in my life.	4.24	1.81
6. Changing my preference from sports to any other recreational activity would require substantial rethinking.	4.07	1.76
*Social bonding*		
7. I enjoy discussing sports with my friends.	4.57	1.87
8. Most of my friends are in some way connected with sports.	4.85	1.72
9. Participating in sports provides me with an opportunity to spend time with friends.	4.48	1.87
*Identity affirmation*		
10. When I participate in sports, I can be myself.	4.59	1.79
11. I resonate with people and media associated with sports.	4.83	1.60
12. When playing sports, I do not have to be concerned about my appearance.	4.89	1.57
*Identity expression*		
13. You can tell a lot about people by seeing them play sports.	4.74	1.68
14. Sport participation says a lot about who I am.	3.81	1.57
15. When I participate in sports, others see me the way I want them to see me.	3.91	1.56

**Table 3:** Summary of Linear Mixed Model.

Effect	Beta	SE	t	*p*
Time	0.731	0.213	3.430	0.001 *
OVSS	0.555	0.434	1.280	0.207
Involvement	1.387	0.415	3.340	0.002 *
Time × OVSS	−0.731	0.296	−2.470	0.017 *
OVSS × involvement	−1.373	0.624	−2.200	0.033 *
Time × involvement	−0.437	0.283	−1.540	0.130
Time × OVSS × involvement	0.987	0.426	2.320	0.024 *

OVSS, online video sport spectatorship; SE, standard error; * *p* < 0.05.

## Data Availability

We can share the dataset.
